# Diverticular Disease Worsening Is Associated with Increased Oxidative Stress and Gut Permeability: New Insights by Circulating Biomarkers

**DOI:** 10.3390/antiox12081537

**Published:** 2023-07-31

**Authors:** Lucia Pallotta, Vittoria Cammisotto, Valentina Castellani, Alessia Gioia, Margherita Spigaroli, Dominga Carlomagno, Simona Bartimoccia, Cristina Nocella, Martina Cappelletti, Stefano Pontone, Roberto Carnevale, Francesco Violi, Rosa Vona, Carla Giordano, Pasquale Pignatelli, Carola Severi

**Affiliations:** 1 Department of Translational and Precision Medicine, Sapienza University of Rome, Viale del Policlinico, 155, 00161 Rome, Italy; alessia.gioia@uniroma1.it (A.G.); margherita.spigaroli@uniroma1.it (M.S.); dominga.carlomagno@uniroma1.it (D.C.); m.cappelletti@uniroma1.it (M.C.); carola.severi@uniroma1.it (C.S.); 2 Department of Clinical, Internal Medicine, Anaesthesiologic and Cardiovascular Sciences, Sapienza University of Rome, Viale del Policlinico, 155, 00161 Rome, Italy; vittoria.cammisotto@uniroma1.it (V.C.); simona.bartimoccia@uniroma1.it (S.B.); cristina.nocella@uniroma1.it (C.N.); francesco.violi@uniroma1.it (F.V.); pasquale.pignatelli@uniroma1.it (P.P.); 3 Department of General Surgery and Surgical Specialty, Sapienza University of Rome, Viale del Policlinico, 00161 Rome, Italy; valentina.castellani@uniroma1.it; 4 Department of Surgery, Sapienza University of Rome, Viale del Policlinico, 155, 00161 Rome, Italy; stefano.pontone@uniroma1.it; 5 Department of Medical-Surgical Sciences and Biotechnologies, Sapienza University of Rome, Corso della Repubblica, 04100 Latina, Italy; roberto.carnevale@uniroma1.it; 6 IRCCS Neuromed, Località Camerelle, 86077 Pozzilli, Italy; 7 Mediterranea Cardiocentro-Napoli, Via Orazio, 80122 Naples, Italy; 8 Center for Gender-Specific Medicine, Istituto Superiore di Sanità, Viale Regina Elena 299, 00161 Rome, Italy; rosa.vona@iss.it; 9 Department of Radiological, Oncological and Pathological Sciences, Sapienza University of Rome, Viale del Policlinico, 155, 00161 Rome, Italy; carla.giordano@uniroma1.it

**Keywords:** human colon, LPS translocation, platelets activation, colonic smooth muscle, antioxidant defense, diverticulosis

## Abstract

Diverticular disease (DD) management is impaired by its pathogenesis, which is still not completely defined, with an unmet clinical need for improved therapies. Ex vivo DD human models demonstrated the presence of a transmural oxidative imbalance that supports an ischemic pathogenesis. This study aimed to assess, with the use of circulating biomarkers, insights into DD pathogenesis and possible therapeutic targets. Nox2-derived peptide, H_2_O_2_, antioxidant capacity, isoprostanes, thromboxanes, TNF-α, LPS and zonulin were evaluated by ELISA in healthy subjects (HS) and asymptomatic and symptomatic DD patients. Compared to HS, DD patients presented low antioxidant capacity and increase in sNox2-dp, H_2_O_2_ and isoprostanes paralleled to a TNFα increase, lower than that of oxidative markers. TxB2 production correlated to Nox2 and isoprostanes, suggesting platelet activation. An increase in zonulin and LPS highlighted the role of gut permeability and LPS translocation in DD pathogenesis. The increase of all the markers statistically correlated with DD severity. The present study confirmed the presence of a main oxidative imbalance in DD and provides evidence of platelet activation driven by LPS translocation. The use of circulating biomarkers could represent a new clinical tool for monitoring disease progression and validate therapeutic strategies never tested in DD as antioxidant supplementation.

## 1. Introduction

Diverticular disease (DD) is one of the most common conditions in Western countries [1] and is a common chronic gastrointestinal condition that places an increasing burden on health-care systems worldwide [2]. DD is a heterogeneous condition with different clinical scenarios and with a prevalence that rises with age, affecting up to two-thirds of people older than 80 years of age [1]. The clinical classification of DD includes asymptomatic diverticulosis (the most frequently occurring at 80% of total DD), symptomatic uncomplicated (15%), and complicated (5%) diverticular disease [3]. Its management is impaired by its pathogenesis, which is still not completely defined, with an unmet clinical need for improved therapeutic strategies. These strategies aim to ease abdominal symptoms and prevent progression and/or relapse of the disease [4]. The pathogenesis of DD is considered to be multifactorial and is still controversially discussed [1]. It is likely to be influenced by the different clinical phenotypes. In the last few decades, significant experimental evidence has accumulated on neuromuscular enteric alterations in DD [5,6]. Alteration of the extracellular matrix is also suggested in diverticulosis and complicated DD by genome-wide association analysis [7,8], whereas evidence of inflammation in DD is still controversial. Studies carried out on small numbers and heterogeneous groups of DD patients support the presence of increased inflammatory infiltration, according to the degree of the disease [9]. However, a recent prospective study did not find an association of colonic diverticula with mucosal inflammation or chronic gastrointestinal symptoms [10].

Although the asymptomatic presence of colonic diverticula, namely diverticulosis, represents the conditio sine qua non for the occurrence of all DD scenarios, it remains unclear if the different clinical phenotypes are part of a continuous development or appear independently [11]. Recent evidence in ex-vivo studies has highlighted the presence of a transmural oxidative imbalance, characterized by an increase in oxidized proteins content and a loss of antioxidants, both in complicated DD (cDD) [12] and diverticulosis [13]. These alterations could be related to the dual pathogenic hypothesis of “traumatic” and “ischemic” mechanisms, proposed for acute diverticulitis [14]. The traumatic damage of a diverticulum by stool impaction may cause acute inflammation, while ischemia may be related to the compression of vascular structures in the neck of the diverticular task, as a result of prolonged and/or recurrent contractile spikes related to neuromuscular alterations in the diverticular tract [15]. Chronic ischemia-reperfusion injuries could then be the culprit for the induction of oxidative stress observed in DD tissues.

Inflammatory cytokines and oxidative stress might have prothrombotic effects on the coagulation cascade through platelet activation that could favor ischemia. Impaired mesenteric vascular endothelial and smooth muscle functions have been reported to be one of the foremost disorders in DD by genome-wide association studies [8]. The issue of the risk of thrombotic complications in chronic gastrointestinal disorders has recently been raised [16], and an incidence rate ratio of 1.36 for thromboembolism has been reported in patients with DD, compared to the general population [17]. Furthermore, a pivotal role in thrombosis may be played by low-grade endotoxemia caused by LPS translocation associated with increased intestinal permeability that affects platelets by shifting them to a procoagulant phenotype that contributes to thrombosis [18]. An increase in permeability has been reported in an ex vivo model of mucosa obtained from symptomatic DD patients [19]. LPS may amplificate the platelet response to common agonists upon interaction with toll-like receptor 4 (TLR4), and via NADPH oxidase-dependent over-overproduction of reactive oxidant species (ROS) and eicosanoids production, including thromboxane A2 (TxA2) and isoprostanes [20].

The aim of this study was to test, by the use of circulating serological markers, the hypothesis that oxidative stress and gut permeability are increased in DD patients with a mechanism involving platelet activation.

## 2. Materials and Methods

A cross-sectional analysis of variables exploring gut permeability, oxidative stress and inflammation was conducted in healthy subjects (HS) (n = 10, 6 males, aged 55.4 ± 2.51 years) and in patients with different DD clinical phenotypes.

(1) Asymptomatic diverticulosis (DIV, n = 10, 7 males, aged 62.4 ± 2.88years): occasional finding of diverticula during screening colonoscopy without symptoms and signs of inflammation;
(2) Symptomatic uncomplicated diverticular disease (SUDD, n = 10, 6 males, aged 64.2 ± 4.35 years): patients with long-lasting/recurrent abdominal pain in the lower abdominal quadrant and changes in bowel habits for at least 3 months with endoscopic evidence of diverticula, without signs of inflammation and without any other causes or symptoms;
(3) Symptomatic complicated diverticular disease (cDD, n = 10, 4 males, aged 55.5 ± 4.63 years): patients with long-lasting/recurrent abdominal pain that develops 3 months after at least one episode of acute diverticulitis.

HS and patients were matched for sex, age and, after enrolment, demographic data regarding their body mass index (BMI) and smoking habits were collected. No statistical differences in age and gender, or in in smoking habits and BMI, were observed between the groups.

Patients were recruited from those referred to the Diverticular Disease Outpatient Clinic of the Gastroenterology Unit of the University Hospital Policlinico Umberto I of Rome from March 2021 to July 2022. Inclusion criteria was age > 18 years and exclusion criteria were the presence of neoplastic or inflammatory bowel disease, hematological diseases, pregnancy, infective or ischemic events 1 month prior blood sampling, the presence of major cardiovascular comorbidities (previous myocardial infarction or stroke) and ongoing antioxidant therapies. The study was approved by the local Ethics Committee (n° 4702, amendment, Protocol code 0496/2022) and written informed consent was obtained according to the principles of the Declaration of Helsinki.

### 2.1. Peripheral Blood Samples and Laboratory Assay

Peripheral blood samples were drawn from HS and patients in fasting conditions, then collected into serum tubes and centrifuged at 300× *g* for 10 min at room temperature. Urine samples were also collected. Serum aliquots were stored at −80 °C for the analysis described below.

### 2.2. Serum sNox2-dp Evaluation

Serum Nox2 was measured as soluble Nox2-derived peptide (sNox2-dp) with an ELISA method, as previously reported [21]. Briefly, the peptide is recognized by binding to a specific monoclonal antibody against the amino acid sequence (224–268) that corresponds to the extracellular membrane part of Nox2 (catalytic core of NADPH oxidase), which was released following platelet activation. The enzyme activity is measured spectrophotometrically by the increased absorbance at 450 nm. Values were expressed as pg/mL; intra-assay and inter-assay coefficients of variation were 8.95% and 9.01%, respectively.

### 2.3. Determination of H_2_O_2_ Production

The hydrogen peroxide (H_2_O_2_) was measured by using a colorimetric assay, as described previously [22]. A standard curve of H_2_O_2_ (0–200 μM) was performed for each assay. Briefly, 50 µL of serum was mixed with 50 µL of 3,3′,5,5′ tetramethylbenzidine in 0.42 mol/L citrate buffer, pH 3.8 and 10 µL of horseradish peroxidase (52.5 U/mL). The samples were incubated at room temperature for 20 min, and the reaction was stopped by the addition of 10 µL 18 N sulphuric acid. The reaction product was measured spectrophotometrically at 450 nm and expressed as μM.

### 2.4. Serum Hydrogen Peroxide Scavenging Activity

To assess the antioxidant capacity, we measured serum hydrogen peroxide break-down activity (HBA) with the HBA assay kit (Aurogene, code HPSA-50). The % of HBA was calculated according to the formula: % Of HBA = [(Ac − As)/Ac] × 100, where Ac is the absorbance of H_2_O_2_ 1.4 mg/mL and as is the absorbance in the presence of the serum sample.

### 2.5. Serum LPS Assay

Serum levels of LPS were measured with a commercial ELISA kit (Cusabio, Houston, TX 77036, USA), according to manufacturer’s instructions. The standards of this kit are LPS purified from Escherichia coli J5. After incubation with a specific antibody, samples were read at 450 nm. Values were expressed as pg/mL; intra-assay and inter-assay coefficients of variation were <10%.

### 2.6. Serum Zonulin Assay

Serum zonulin levels were measured with a commercial ELISA kit (Elabscience, Huston, TX, USA). Standards and samples were incubated 90 min at 37 °C into a microplate pre-coated with a specific antibody for zonulin. The amount of zonulin was measured with a microplate auto-reader at 450 nm. Values were expressed as ng/mL; intra-assay and inter-assay coefficients of variation were <10%.

### 2.7. 8-Iso-PGF 2α Assay

Serum and urinary isoprostanes (8-iso-PGF2α) were measured with a commercially available ELISA kit (Elabscience, Houston, TX, USA) and the values were expressed in pg/mL. Intra-assay and inter-assay coefficients of variation were both <10%.

### 2.8. TNF-α Assay

The blood levels of pro-inflammatory cytokine TNF-α was evaluated with an ELISA kit (Diaclone, Besançon, France) according to the manufacturer’s instructions. The TNF-α levels in the samples were established by comparison of the optical density (OD) of the samples with the standard curve OD. The values were expressed as pg/mL; intra- and inter-assay CV were <10%.

### 2.9. Thromboxane Assay

Serum TxA2 was analyzed by estimating its stable metabolite, named TxB2, in the serum with an ELISA commercial kit (Cusabio, Houston, TX, USA), according to manufacturer’s instructions. The values were expressed as pg/mL. Intra- and inter-assay coefficients of variation for TxB2 were <8% and <10%, respectively.

### 2.10. Statistical Analysis

MedCalc Statistical Software (MedCalc, Ostend, Belgium) was used for statistical analysis. Data are expressed as mean ± standard error (SE). *p* value < 0.05 is considered statistically significant. To evaluate if variables have a normal distribution, a Shapiro test was executed. The analysis of difference between groups was obtained one-way ANOVA for variables normally distributed and Kruskal–Wallis test for variables not normally distributed.

## 3. Results

Circulating levels of soluble NOX2-derived peptide (sNOX2-dp)—a direct marker of NADPH oxidase activation mainly produced by activated platelets [23]—H_2_O_2_ and isoprostane production—as well as antioxidant capacity, evaluated by HBA—were assessed. This was carried out in order to establish the possible involvement of oxidative stress in DD.

**Figure 1:**
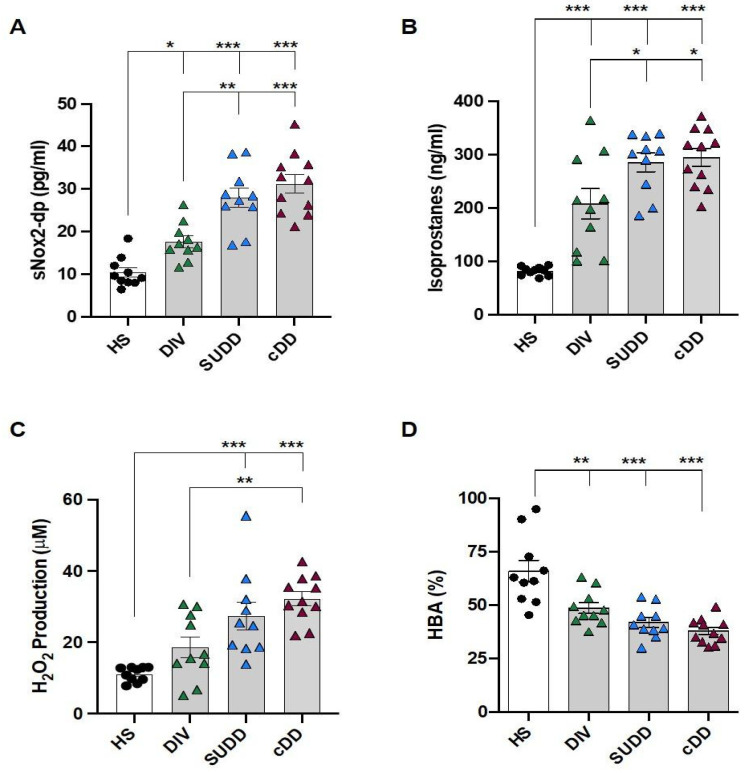
Oxidative circulating biomarkers. Bar graphs (white: healthy subjects; grey: diverticular disease patients) show serum levels of (**A**) soluble Nox2–derived peptide (sNox2-dp); (**B**) isoprostanes; (**C**) H_2_O_2_; and (**D**) hydrogen peroxide breakdown activity (HBA). HS: healthy subjects, DIV: asymptomatic diverticulosis, SUDD: symptomatic uncomplicated diverticular disease; cDD: symptomatic complicated diverticular disease. Data are expressed as mean ± SE, * *p* < 0.05, ** *p* < 0.01, *** *p* < 0.001. *p* values were calculated using one-way ANOVA with post-hoc Tukey correction.

Overall, the DD patients presented higher levels of sNox2-dp, H_2_O_2_ and isoprostane production and a lower antioxidant capacity compared to HS (Figure 1A–D). The urinary isoprostanes reflected the same trend observed in serum (data not shown).

A significant difference in oxidative stress parameters was observed across DD groups (Figure 1A–D). In particular, sNox2-dp and serum isoprostane levels significantly differed from asymptomatic and symptomatic DD (Figure 1A,B).

The increase of all oxidative circulating biomarkers resulted associated with severity of DD (Table 1).

### 3.1. Role of Inflammation in Diverticular Disease

**Figure 2:**
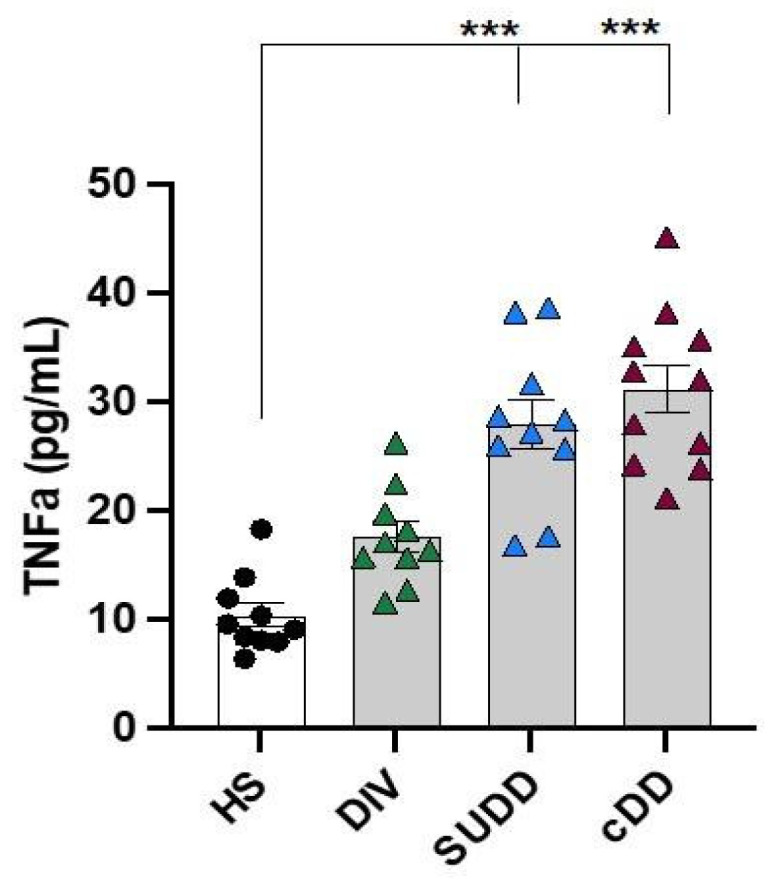
Inflammatory biomarker. Bar graphs (white: healthy subjects; grey: diverticular disease patients) show serum levels of TNFα. HS: healthy subjects; DIV: asymptomatic diverticulosis; SUDD: symptomatic uncomplicated diverticular disease; cDD: symptomatic complicated diverticular disease. Data are expressed as mean ± SE, *** *p* < 0.001. *p* values were calculated using Kruskal–Wallis test with post-hoc Dunnett’s correction.

Circulating levels of TNFα were also measured in DD patients in order to define the presence of systemic inflammation. Higher levels of this pro-inflammatory cytokine in symptomatic DD patients were found compared to HS (Figure 2), which progressively increased with the severity of DD (r = 0.72, 95% CI 0.53–0.84, *p* < 0.0001), similar to oxidative parameters.

However, in respect to HS, the increase in TNFα in cDD patients was statistically lower than that of isoprostanes in all groups of patients (DIV: 1.39 ± 0.08 vs. 2.53 ± 0.35, *p* < 0.001; SUDD: 2.04 ± 0.19 vs. 3.46 ± 0.21, *p* < 0.001; cDD: 2.03 ± 0.11 vs. 3.57 ± 0.20, *p* < 0.001). The increase of H_2_O_2_ was statistically higher than TNFα only in CDD (2.92 ± 0.18 vs. 2.03 ± 0.11, *p* < 0.001).

### 3.2. Role of Gut Permeability in Diverticular Disease

**Figure 3:**
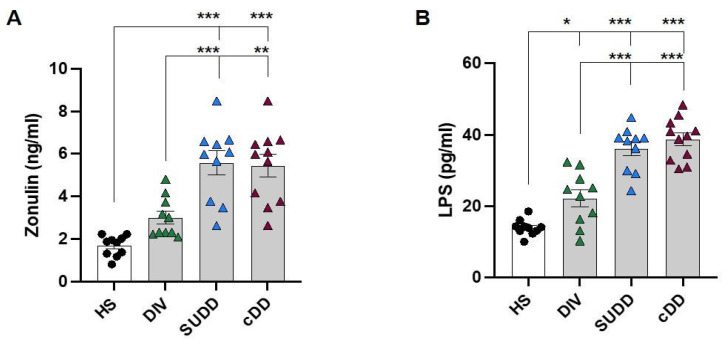
Intestinal permeability and LPS. Bar graphs (white: healthy subjects; grey: diverticular disease patients) show serum levels of (**A**) zonulin and (**B**) circulating LPS. HS: healthy subjects; DIV: asymptomatic diverticulosis; SUDD: symptomatic uncomplicated diverticular disease; cDD: symptomatic complicated diverticular disease. Data are expressed as mean ± SE, * *p* < 0.05, ** *p* < 0.01, *** *p* < 0.001. *p* values were calculated using one-way ANOVA with post-hoc Tukey correction.

The pathogenic role of gut permeability and LPS translocation in DD pathogenesis, were evaluated (Figure 3A,B). Serum zonulin and LPS were higher in patients with DD than in HS, and progressively increased in association with the degree of DD severity.

Both parameters were statistically higher in symptomatic patients with respect to asymptomatic diverticulosis. LPS significantly correlated with zonulin (r = 0.58, 95% CI: 0.33–0.75, *p* < 0.0001). Notably, a positive linear correlation was found between LPS both with sNOX2-dp (r = 0.78, 95% CI: 0.62–0.88, *p* < 0.0001) and TxB2 (r = 0.79, 95% CI: 0.64–0.88, *p* < 0.0001), suggesting that gut-derived LPS might be responsible for platelet activation observed in DD patients.

### 3.3. Platelet Activation in Diverticular Disease

**Figure 4:**
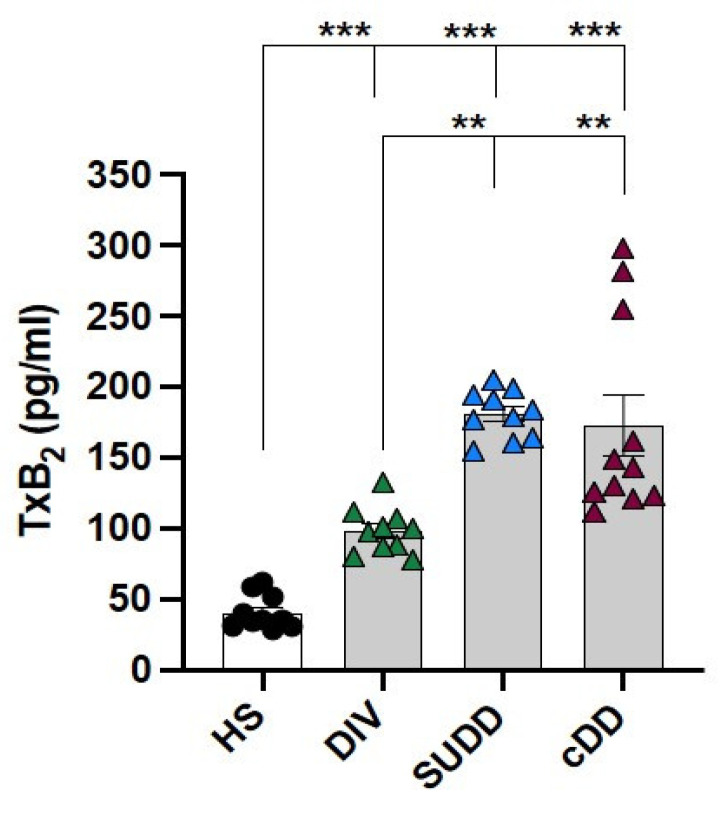
Platelet activation biomarker. Bar graphs (white: healthy subjects; grey: diverticular disease patients) show serum levels of thromboxane A2 (TxB_2_). HS: healthy subjects; DIV: asymptomatic diverticulosis; SUDD: symptomatic uncomplicated diverticular disease; cDD: symptomatic complicated diverticular disease. Data are expressed as mean ± SE, ** *p* < 0.01, *** *p* < 0.001. *p* values were calculated using Kruskal–Wallis test with post-hoc Dunnett’s correction.

Finally, in order to assess if the increase in circulating levels of sNOX2-dp and isoprostanes might be correlated to platelet activation in DD patients, TxB2 production was assessed. A statistical progressive increase in TxB2 levels was observed in DD patients, compared to HS, and that result was associated with the severity of DD (r = 0.78, 95% CI: 0.63–0.88, *p* < 0.0001) (Figure 4).

## 4. Discussion

This pilot study opens new insights into the pathogenesis of DD and identifies quantitative non-invasive and reliable circulating biomarkers that could be of support in future clinical studies to monitor treatment efficacy on both symptoms control and disease progression. Notably, the progressive significant increase observed in all the markers, from asymptomatic diverticulosis to symptomatic diverticular disease, suggests that DD phenotypes are not distinct diseases, but different stages of a progressive decrease [4].

The present results maintain that both oxidative stress [24] and inflammatory mechanisms [25] are relevant in DD pathogenesis. The reported circulating increase in oxidative stress biomarkers paralleled to a decrease in systemic antioxidant capacity are in accordance with recent observations obtained from ex vivo human models of DD that strengthen the predominant role of oxidative imbalance in the pathogenesis of this disease [12]. Ex vivo human studies on complicated DD highlighted the presence of a prevalent transmural oxidative imbalance characterized by a homogenous increase in oxidative stress markers through all the wall layers with loss in antioxidant defenses, without a peculiar increase in inflammatory markers. Oxidative stress might manifest downstream of inflammation or other medical complications (ischemia-reflow). On the basis of available data, the main probable hypothesis is that oxidative imbalance might be related to chronic local ischemia-reperfusion injury that impaired colonic smooth muscle activity and mesenteric vasculature, leading to a chronic consumption of antioxidant molecules. The trigger of ischemia could be recurrent transient compressions of vasa recta in the small “neck” of the diverticulum, caused by a marked contractile spike of the colon [14]. However, inflammation also contributes to the oxidative imbalance, albeit to a lesser extent, as evident with the observed increase in circulating TNF-α in symptomatic DD, a pleiotropic inflammatory cytokine known for its involvement in ROS production [26]. Also, previous studies on circulating IL-6 showed a correlation between chronic systemic inflammation and the risk of diverticulitis [27].

The growing significant increase of all the markers from asymptomatic diverticulosis to symptomatic diverticular disease, without differences between uncomplicated (SUDD) and complicated DD, suggests that the onset of symptoms might be triggered by the worsening of these biochemical alterations. The emerging pathogenic role of oxidative imbalance that characterizes DD might be an important new therapeutic target never tested in this disease. Recent evidence shows that, with respect to healthy subjects, DD patient diet has a reduced oxygen radical absorbance capacity and a lower antioxidant content [28]. The main probable hypothesis is that oxidative imbalance might be related to chronic local ischemia-reperfusion injury that impaired colonic smooth muscle activity and mesenteric vasculature.

In this setting, the role of LPS translocation and alterations in gut permeability in DD is notable, and is suggested by the increase in zonulin, an indirect measure of gut permeability [29]. The role of these two events in pathogenesis of DD has been already hypothesized [4,19,30]. The increase in gut permeability might be related to both an overgrowth of gut bacteria inside the diverticula and to the thin diverticula wall. LPS translocation might have a dual role in DD pathogenesis. Its presence in the perivisceral environment might activate myogenic TLR4 [31] that are capable of triggering persistent and long-term oxidation-driven phenotypic myogenic cellular alterations [32], favoring the hypercontractile state that characterizes this disease [11]. These perivisceral effects could be responsible for the main involvement of longitudinal muscle in tissue alterations observed in ex vivo DD models [12]. In addition, LPS may amplificated platelet response to common agonists upon interaction with toll-like receptor 4 (TLR4) and via the overproduction of reactive oxidant species (ROS) and eicosanoids, including thromboxane A2 (TxA2) and isoprostanes [20]. In particular, the increase in Nox2, the catalytic core of nicotinamide adenine dinucleotide phosphate (NADPH) oxidase—which is among the most important cellular producer of platelet ROS and is implicated in platelet activation [22]—that correlated to the increase in production of eicosanoids, isoprostanes and thromboxane, suggest a possible role of platelet activation. This could contribute to the oxidative burden and to transient ischemia. Platelet hyperreactivity is supported by the increase in LPS and TNFα, two known triggers of Nox2 [18,33,34] observed in DD patients. Isoprostanes are considered as the most reliable markers of oxidative stress in a number of human pathologies [35]. In addition to being markers of oxidative stress, isoprostanes appeared to be the mediators of important biological effects such as stimulation of collagen synthesis [36], another hallmark of DD [5].

The circulating biomarkers identified with this pilot study could represent a new clinical tool aimed to monitor the course of the disease and to validate therapeutic strategies. To date, the main available biomarkers for DD have focused on the severity of acute diverticulitis, namely on their ability to predict a complicated course of acute diverticulitis, the best being C-Reactive [37,38]. As for C-reactive protein, the other serological markers that have been investigated (white blood cells count, erythrocyte sedimentation rate, bilirubin, alkaline phosphatase, α1-acid glycoprotein) were focused on assessing and monitoring the course of acute diverticulitis [39]. None of these markers, which included several cytokines [40], resulted in the useful clinical management of overall DD clinical phenotypes, since no specific alterations were reported in patients with SUDD, decreasing their clinical usefulness as a therapeutic marker of DD. The only promising tool proposed for the response to the therapeutic treatment and for the prediction of diverticulitis recurrence is fecal calprotectin [41], which is useful in distinguishing patients affected by symptomatic DD or irritable bowel syndrome DD [37]. This marker increases in SUDD patients and decreases following therapy [42]. However, recent German guidelines on diverticular disease/diverticulitis do not recommend its use [43]. Calprotectin specificity is influenced by several factors, such as non-inflammatory gastrointestinal conditions, medication and lifestyle [44].

## 5. Conclusions

The present study provides two important clinical implications. First, the circulating biomarkers identified with this pilot study could represent a new clinical tool to monitor the course of the disease and to validate therapeutic strategies. Collectively, our data also displayed evidence of an increase in platelet activation in DD patients that could exhibit an increased risk of cardiovascular complications, similar to other chronic gastrointestinal disorders. Thus, improving platelet activation oxidative stress-derived could be used in secondary prevention, leading to the reduction of potential thrombotic complications.

## Figures and Tables

**Table 1:** Correlation between severity of diverticular disease and oxidative circulating biomarkers.

DD Severity vs.	Correlation Coefficient r	Significance Level	95% Confidence Interval for r
**sNOX2-dp**	0.81	*p* < 0.0001	0.68 to 0.89
**Isoprostanes**	0.78	*p* < 0.0001	0.62 to 0.88
**H_2_O_2_**	0.71	*p* < 0.0001	0.52 to 0.84
**HBA**	−0.69	*p* < 0.0001	−0.83 to −0.49

## Data Availability

The data presented in this study are available on request from the corresponding authors.
